# Significant changes in the practice of chest radiography in Dutch intensive care units: a web-based survey

**DOI:** 10.1186/2110-5820-4-10

**Published:** 2014-04-04

**Authors:** Martijn Tolsma, Tom A Rijpstra, Marcus J Schultz, Paul GH Mulder, Nardo JM van der Meer

**Affiliations:** 1 Department of Intensive Care, Amphia Hospital, Breda, Oosterhout, The Netherlands; 2 Department of Intensive Care, University Medical Center, Postbus 85500, 3508 GA Utrecht, The Netherlands; 3 Department of Intensive Care, Academic Medical Center, Amsterdam, The Netherlands; 4 Tias Nimbas Business School, Tilburg University, Tilburg, The Netherlands; 5 Amphia Academy, Amphia Hospital, Breda, Oosterhout, The Netherlands

**Keywords:** Chest radiography, Imaging, Intensive care

## Abstract

**Background:**

ICU patients frequently undergo chest radiographs (CXRs). The diagnostic and therapeutic efficacy of routine CXRs are now known to be low, but the discussion regarding specific indications for CXRs in critically ill patients and the safety of abandoning routine CXRs is still ongoing. We performed a survey of Dutch intensivists on the current practice of chest radiography in their departments.

**Methods:**

Web-based questionnaires, containing questions regarding ICU characteristics, ICU patients, daily CXR strategies, indications for routine CXRs and the practice of radiologic evaluation, were sent to the medical directors of all adult ICUs in the Netherlands. CXR strategies were compared between all academic and non-academic hospitals and between ICUs of different sizes. A comparison was made between the survey results obtained in 2006 and 2013.

**Results:**

Of the 83 ICUs that were contacted, 69 (83%) responded to the survey. Only 7% of responding ICUs were currently performing daily routine CXRs for all patients, and 61% of the responding ICUs were said never to perform CXRs on a routine basis. A daily meeting with a radiologist is an established practice in 72% of the responding ICUs and is judged to be important or even essential by those ICUs. The therapeutic efficacy of routine CXRs was assumed by intensivists to be lower than 10% or to be between 10 and 20%. The efficacy of ‘on-demand’ CXRs was assumed to be between 10 and 60%. There is a consensus between intensivists to perform a routine CXR after endotracheal intubation, chest tube placement or central venous catheterization.

**Conclusion:**

The strategy of daily routine CXRs for critically ill and mechanically ventilated patients has turned from being a common practice in 2006 to a rare current practice. Other routine strategies and an ‘on-demand only’ strategy have become more popular. Intensivists still assume the value of CXRs to be higher than the efficacy that is reported in the literature.

## Background

ICU patients frequently undergo chest radiographs (CXRs) on a routine basis, after a change in their clinical situation or directly after surgery. Several investigators have studied the clinical value of routine CXRs following central venous catheterization, endotracheal intubation, cardiac surgery, pulmonary surgery or chest tube placement and removal [1-18]. Other investigators have studied the value of daily routine CXRs in a mixed ICU population or in mechanically ventilated patients only [19-28]. The diagnostic and therapeutic efficacy of these routine radiographs is now known to be low [1-3,6-10,12-15,17,19,20,22-25,28]. Studies that compared a routine CXR strategy with an ‘on-demand’ CXR strategy did not show any difference in outcome measures [29-34].

Despite these results, in 2006, Graat *et al*. showed that in a majority of ICUs in The Netherlands, a daily routine CXR strategy was still common practice [35]. Intensivists at that time assumed a higher value of daily CXRs than had been reported in the literature. Although a more restrictive CXR strategy seems safe, Ganapathy *et al*. stated in a more recent meta-analysis that study populations were small and the number of missed findings had not been sufficiently evaluated [33]. Meanwhile, the discussion regarding specific indications for CXRs in critically ill patients and the safety of abandoning routine CXRs is still ongoing. We performed a new survey among Dutch intensivists on their current chest radiography practice in order to study the influence of time and knowledge in relation to any changes in that practice.

## Methods

For our study, we selected all Dutch academic hospitals (related to a university medical school) and non-academic hospitals with an independent adult ICU department. A web-based questionnaire (Additional file 1), deployed using the website http://www.thesistools.com, was sent to the medical staff of these ICUs by the end of April 2013. A reminder was sent after two weeks, four weeks and six weeks after the questionnaire was originally sent. All hospitals received one questionnaire, as it is currently common in our country to have one adult ICU center with a combined medical staff and a mixed patient population. For data analysis, we included all questionnaires that were answered within eight weeks from the start of the study, with a response of more than 80%. The questionnaire we deployed was based on the questionnaire used previously in 2006 [35]. After confirming a specific hospital’s response, this response was made anonymously. To prevent duplicated data, additional responses from the same hospital were not included.

The survey contained questions regarding hospital and ICU characteristics, type of ICU patients, CXR strategies, indications for routine CXRs and the practice of radiologic evaluation. Regarding ICU size, only beds with the possibility of mechanical ventilation were taken into account. We asked the intensivists to judge the clinical value (therapeutic efficacy) of routine and ‘on-demand’ CXRs and to judge the value of an established radiologic evaluation with a radiologist. We finally asked them to state some indications for routine CXRs.

CXR strategies were compared between all hospitals, between academic and non-academic hospitals and between ICUs of different sizes. A comparison was made between the survey results from 2006 and 2013. Therapeutic efficacy was defined as the percent of CXR findings that resulted in a subsequent change in patient management.

Data analysis was performed using IBM SPSS Statistics 21.0 (IBM,Armonk, NY, USA). All variables were expressed as counts (%). Differences in CXR strategies between 2006 and 2013 were examined using Fisher’s exact test.

## Results

A total of 83 hospitals with an adult ICU were selected for this study, and 69 hospitals (83%) responded to the web-survey. The non-responders were one academic hospital and thirteen non-academic hospitals of limited size. The characteristics of the responding ICU departments are shown in Table 1. Only 10% of the responders were academic hospitals, while 90% of the institutions were non-academic. Most ICUs (58%) had between five and fifteen beds with the option of mechanical ventilation available, and 29% of ICUs had more than fifteen beds with the option of mechanical ventilation available. The most frequent number of fulltime intensivists available was one to five (46%) or five to ten (36%). Cardiac surgery patients were admitted to 29% of the responding ICUs, and neurosurgical patients were admitted to 23% of the responding ICUs.

**Table 1:** Hospital and ICU characteristics (All hospitals, n = 69)

**Hospital type; n (%)**	
Academic	7 (10)
Non-academic	62 (90)
ICU level; n (%)	
Level 1^1^	25 (36)
Level 2^2^	18 (26)
Level 3^3^	26 (38)
Number of ICU beds; n (%)	
< 5	9 (13)
5 to 15	40 (58)
> 15	20 (29)
Number of fulltime intensivists; n (%)	
1 to 5	32 (46)
5 to 10	25 (36)
11 to 20	12 (17)

n = number.

^1^Intensivist available in hospital on weekdays during daytime; 2.7 fulltime ICU nurses per bed.

^2^Intensivist exclusively available for ICU on seven days a week during daytime; 3.5 fulltime ICU nurses per bed.

^3^Intensivist exclusively available for ICU on seven days a week during day and night; 4.2 fulltime ICU nurses per bed.

Of all hospitals, 39% practiced some kind of routine CXR strategy, but only 7% of ICUs obtained daily routine CXRs for all patients (Table 2). Some other ICUs only performed daily routine CXRs for mechanically ventilated patients (6%), patients in the first days of ICU admission (4%), all patients on certain fixed days of the week (3%) or for cardiothoracic surgery patients only (6%). Most ICU departments (61%) state that they never perform daily CXRs on a routine basis. A distinctive group seems to be the academic ICUs and largest non-academic ICUs, because 86% of the academic ICUs and 75% of the ICUs with > 15 beds practice some kind of routine chest radiography strategy

**Table 2:** Current CXR practice

	**Routine strategy**	**‘On-demand only’**
All hospitals (n = 69); n (%)	27 (39)	42 (61)
All patients	5 (7)	-
Patients on ventilation only	4 (6)	-
Certain fixed days a week	3 (4)	-
First days of admission only	2 (3)	-
Cardiothoracic surgery patients only	4 (6)	-
Other, not specified	9 (13)	-
Academic hospitals (n = 7); n (%)	6 (86)	1 (14)
Non-academic hospitals (n = 62); n (%)	21 (34)	41 (66)
ICU < 5 beds (n = 9); n (%)	3 (33)	6 (67)
ICU 5 to 15 beds (n = 40); n (%)	9 (22)	31 (78)
ICU > 15 beds (n = 20); n (%)	15 (75)	5 (25)

CXR = chest radiograph; n = number.

Table 3 presents a comparison of the survey results from 2006 and the results of the current study. The number of ICUs that used some kind of routine CXR strategy decreased from 63 to 39% from 2006 to 2013 (*P* = 0.018). There was a decrease in the use of a daily routine CXR strategy for all ICU patients although this decrease was not significant (*P* = 0.324). However, there was an important decrease in the use of a routine CXR strategy for mechanically ventilated patients (*P* < 0.001). The frequency of other routine strategies and, in particular, of an ‘on-demand only’ strategy increased from 2006 to 2013 (*P* = 0.095 and *P* = 0.018). There were no significant differences in the performance of routine CXRs after chest tube placement, endotracheal intubation, central venous catheterization, cardiopulmonary resuscitation or tracheostomy.

**Table 3:** Comparison of CXR strategies between 2006 and 2013

	**2006 (n = 41)**	**2013 (n = 69)**	***P*-value**
Daily routine CXR strategy; n (%)	26 (63)	27 (39)	0.018
All patients	6 (15)	5 (7)	0.324
Mechanically ventilated patients	15 (37)	4 (6)	< 0.001
Other daily routine strategy	5 (12)	18 (26)	0.095
‘On-demand only’ strategy; n (%)	15 (37)	42 (61)	0.018
Routine CXR after; n (%)			
Chest tube placement	40 (98)	68 (99)	1.000
Endotracheal intubation	31 (76)	53 (77)	1.000
CVL placement	34 (83)	52 (76)	0.475
CPR setting	24 (59)	40 (68)	1.000
Tracheostomy	24 (59)	30 (43)	0.168

CPR = cardiopulmonary resuscitation; CVL = central venous line; CXR = chest radiograph.

The practices of radiologic evaluation with a radiologist are shown in Table 4. The majority of ICUs had a daily established meeting with a radiologist, and this daily meeting also included weekend days for 28% of ICUs and were on weekdays only for 44% of ICUs. Only 12% of the responding ICU departments never evaluate their CXRs in a specially arranged meeting. A daily radiological conference was considered essential by 46% of the ICUs and good for cooperation between medical staff by 74% of the ICUs. The training purposes of a daily radiologic conference were considered important by only 19% of the ICUs.

**Table 4:** Practice of radiologic evaluation (All hospitals, n = 69)

**Radiologic conference; n (%)**	
Daily	19 (28)
Daily except weekends	30 (44)
On request only	12 (17)
Never	8 (12)
Judged value of radiologic conference; n (%)	
Worthless	6 (9)
Essential	32 (46)
Good for cooperation	51 (74)
Required for training purpose	13 (19)

n = number.

Table 5 shows the responding intensivists’ assumed therapeutic efficacy values for CXRs performed routinely and CXRs performed on a special indication only (‘on-demand’). The efficacy of routine CXRs was generally assumed to be lower than 10% or to be between 10 and 20%. The efficacy for ‘on-demand’ CXRs was obviously assumed to be higher, somewhere between 10 and 60%.

**Table 5:** Assumed efficacy value of CXRs (All hospitals, n = 69)

**Assumed therapeutic efficacy; n (%)**	**Routine CXR**	**‘On-demand’CXR**
< 10%	17 (25)	5 (7)
10 to 20%	11 (16)	21 (30)
20 to 30%	6 (9)	23 (33)
30 to 60%	3 (4)	17 (25)
> 60%	0 (0)	3 (4)
Not applicable	32 (46)	

CXR = chest radiograph; n = number.

There seems to be a consensus for the indication of a routine CXR after chest tube placement and central venous catheterization (Table 6). Other frequently suggested indications for CXRs are the diagnostic workups for the presence of a pneumothorax, pneumonia or adult respiratory distress syndrome (ARDS).

**Table 6:** Suggested indications for which a CXR is deemed essential for diagnosis or assessment (All hospitals, n = 69)

**Indication; n (%)**	
Presence of ARDS	43 (62)
Presence of a pneumonia	47 (68)
Presence of a pneumothorax	53 (77)
Patients volume status	12 (17)
Correct position of CVL	64 (93)
Correct position of chest tube	66 (96)
Correct position of IABP	36 (52)

ARDS = Adult Respiratory Distress Syndrome; CVL = central venous line; CXR = chest radiograph; IABP = intra-aortic balloon pump; n = number.

## Discussion

Our results show that a strategy of daily routine CXRs is performed for all patients in only 7% of ICUs and for all mechanically ventilated patients in only 6% of ICUs, while 61% of the ICUs never perform CXRs on a routine basis. A daily meeting with a radiologist is an established practice in the majority of ICUs and is judged to be important or even essential. Our results are in line with the results of Lakhal *et al*. who did an observational day study in French ICUs in 2010 [36]. In their study population, a daily routine CXR strategy was also practiced in 7% of ICUs, while 63% of ICUs never performed routine CXRs. Compared to the results of Graat *et al*. in 2006, there has been an obvious change in chest radiography strategies in Dutch ICUs. Then, the majority of ICUs practiced a daily routine CXR strategy [35]. An ‘on-demand only’ strategy and other more liberal routine strategies have become more common in recent years.

The indications for routine CXRs suggested by the responders in our survey are, in general, comparable to the indications suggested in the surveys performed by Graat *et al*. and Hejblum *et al*. [35,36]. There is still consensus between intensivists regarding the importance of obtaining a CXR after endotracheal intubation, chest tube placement and central venous catheterization and for diagnostic workups for pneumonia, ARDS or pneumothorax. However, the indications for a routine CXR after intubation and central venous catheterization are not supported by the literature [1-3,6]. There is no consensus that a routine CXR should be performed for all mechanically ventilated patients [35,37].

Although our results, and the reduction in routine CXR strategies, suggest that intensivists seem to be aware of the limited clinical value of routine CXRs, they still assume this value to be higher than the efficacy that is reported in the literature [35]. This is also true for the clinical value of ‘on-demand’ CXRs. In recent literature, the reported diagnostic efficacy for CXR small findings is between 30 and 65%, while the diagnostic efficacy for important findings and the therapeutic efficacy of CXRs are reported to be between 2 and 7% [22-24,28]. Intensivists may assume a higher clinical value of CXRs due to the value of negative CXR findings, which has not been previously studied. The ability of CXR findings to exclude complications, certain clinical situations or the need for an intervention, probably has a clinical impact that is hard to study.

During the last decade, multiple studies have shown that an ‘on-demand’ CXR strategy increases the diagnostic and therapeutic efficacy of CXRs in critically ill patients while subsequently reducing the number of CXRs and subsequent costs significantly. No difference in mortality, length of mechanical ventilation or length of ICU or hospital stay was found [29-34]. Kroner *et al*. found no change in the number of computed tomography (CT) or ultrasound studies performed by the department of radiology for ICUs that use an ‘on-demand’ CXR strategy [34]. To our knowledge there are no studies regarding the impact of an ‘on-demand’ CXR strategy on the number of ultrasound studies performed by intensivists or *vice versa*. A routine ultrasound examination of the pleura and pericardium performed by ICU physicians after cardiac surgery or before ICU discharge might further reduce the use routine CXR strategies.

However, completely abandoning routine CXRs for ICU patients is still under debate because the currently available studies did not evaluate the effect of missed findings, had low patient numbers and did not rigorously assess possible harm [33]. More prospective studies need to be performed on the topic of missed findings, the clinical value of negative findings and the indications for CXRs in an ‘on-demand only’ strategy, before a definitive conclusion can be drawn.

## Conclusions

The strategy of daily routine CXRs for critically ill and mechanically ventilated patients has turned from being a common practice in 2006 to a rare current practice. Other routine strategies and an ‘on-demand only’ strategy have become more popular. Intensivists still assume that the value of CXRs is higher than the efficacy reported in the literature.

## Abbreviations

ARDS: Adult Respiratory Distress Syndrome; CPR: Cardiopulmonary resuscitation; CT: Computed tomography; CVL: Central venous line; CXR: Chest radiograph; CXRs: Chest radiographs; IABP: Intra-aortic balloon pump; ICU: Iintensive care unit.

## Competing interests

All authors declare that they have no competing interests.

## Authors’ contributions

MT participated in the study design, data acquisition, data analysis, data interpretation and the writing of the manuscript. TR and MS participated in the study design and manuscript revision. PM participated in the data analysis and manuscript revision. NM participated in the study design, data analysis, data interpretation and the writing and revising of the manuscript. All authors have read and approved the final manuscript.

## Supplementary Material

Additional file 1 Websurvey Chest Radiography Practice in Dutch Intensive Care Units. Click here for file
