# Supplementary material for: Significant changes in the practice of chest radiography in Dutch intensive care units: a web-based survey
Source: Ann Intensive Care. 2014 Apr 4;4:10. doi: 10.1186/2110-5820-4-10 (PMC4113284; doi:10.1186/2110-5820-4-10)
Supplement: Additional file 1 — Websurvey Chest Radiography Practice in Dutch Intensive Care Units. [file 2110-5820-4-10-S1.pdf]

Appendix 1.

Questionnaire.

1. In what type of hospital do you work?

a. Academic Hospital

b. Non-academic Hospital

2. What is your Intensive Care Unit (ICU) level?

a. Level 1

b. Level 2

c. Level 3

3. How many ICU beds do you have available for mechanical ventilation?

a. <5 beds

b. 5-15 beds

c. > 15 beds

4. How many full time intensivists are there available?

a. 1-5

b. 5-10

c. 10-20

d. >20

5. Which type of patients are admitted on the ICU? (more than one answer is possible)

a. Medical patients

b. Surgical patients

c. Cardiosurgical patients

d. Neurosurgical patients

6. For which patients do you perform a daily routine chest radiograph (CXR)? (more than one answer possible)

a. For no patient, we do not perform daily routine CXRs

b. For mechanically ventilated patients only

c. For all patients

d. Other strategy; specify………

7. For which of the following procedures do you perform a CXR routinely? (more than one answer possible)

a. After endotracheal intubation

b. After central venous catheterisation

c. After pulmonary artery catheter placement

d. After tracheostomy placement

e. After chest tube placement

f. After intra aortic balloon pump (IABP) placement

g. After a cardiopulmonary resuscitation (CPR) setting

h. After a change in ventilator settings

i. After arrival of a postsurgical patient

j. Before extubation

k. After extubation

l. Before ICU discharge

8. For which part of cases do you think that the CXR findings do change patient management when the CXR is performed routinely?

a. <10%

b. 10-20%

c. 20-30%

d. 30-60%

e. >60%

f. We never perform routine CXRs

9. For which part of cases do you think that the CXR findings do change patient management when the CXR is performed on indication (on-demand)?

a. <10%

b. 10-20%

c. 20-30%

d. 30-60%

e. >60%

10. For which indications do you think a CXR is essential for diagnosis or assessment? (more than one answer possible)

a. Presence of an acute respiratory distress syndrome (ARDS)

b. Presence of a pneumonia

c. Presence of a pneumothorax

d. Patients volume status

e. Correct placement of a central venous catheter

f. Correct placement of a chest tube

g. Correct placement of an IABP

11. How do you judge a daily established radiologic conference? (more than one answer possible)

a. Worthless

b. Essential

c. Good for cooperation

d. Important for training purposes

12. When do you have an established radiologic conference?

a. Daily, including the weekends

b. Daily, except the weekends

c. On request only

d. There is no established meeting
